# A Scoping Review of Outcome Measurement Instruments Assessing Healthcare Professionals’ Knowledge, Attitudes and Beliefs About Adult Obesity

**DOI:** 10.1155/jobe/1363752

**Published:** 2026-08-13

**Authors:** Paul MacDonald, Derek Peters, Helen Frank, Rebecca Stack

**Affiliations:** ^1^ School of Allied Health and Community, University of Worcester, Worcester, England, UK, worcester.ac.uk

## Abstract

**Background:**

The relationship between knowledge, attitudes and beliefs (KAB) is complex and reciprocal. Although KAB are internal constructs, they collectively influence behaviour without necessarily determining it. In obesity management, understanding healthcare professionals’ (HCPs’) KAB about adult obesity is crucial. However, comprehensive and valid outcome measurement instruments (OMIs) are necessary to accurately reflect their perspectives.

**Objective:**

This scoping review aimed to identify and evaluate published OMIs that assess HCPs’ KAB about adult obesity, utilising the Consensus‐based Standards for the Selection of Health Measurement Instruments (COSMIN) framework.

**Methods:**

Following the COSMIN methodology and the five‐stage scoping review framework proposed by Arksey and O’Malley, and refined by Levac and Colquhoun, reviewers searched six databases—Embase, Medline, Web of Science, PsycINFO, CINAHL and Google Scholar—for studies published from 1 January 1991 to 1 May 2025. Inclusion criteria required studies to focus on HCPs’ KAB, OMI development, psychometric evaluation or application in relation to adult obesity. Two reviewers screened the literature and assessed methodological quality using the COSMIN Risk of Bias checklist, and evidence quality was graded with a modified GRADE approach.

**Results:**

A total of 9213 records were identified, 45 articles were evaluated for full‐text eligibility, and 4 articles were selected for final review. According to COSMIN criteria, the methodological quality of these studies was deemed inadequate. None demonstrated sufficient content validity, with common limitations including unrepresentative samples of HCPs’ and poorly defined constructs. As per the COSMIN ‘stop rule’,” no further psychometric evaluations were conducted.

**Conclusion:**

There is a significant gap in the availability of rigorously developed instruments for assessing HCPs’ KAB about adult obesity. There is a clear need for more contemporary and reliable OMIs that adhere to quality standards, such as those outlined in the COSMIN framework.

## 1. Introduction

The relationship between knowledge, attitudes, and beliefs (KAB) has long been debated. Early research questioned whether knowledge directly influences behaviour [1], while later studies emphasised that this relationship is complex and reciprocal [2, 3]. Underlying beliefs shape how individuals respond to new information and develop attitudes. Although KAB are internal constructs, they collectively influence behaviour without necessarily determining it. Therefore, stating that a healthcare professional (HCP) possesses accurate knowledge or has a favourable attitude does not guarantee a change in behaviour [4].

This review explores the constructs of KAB, which are interrelated yet distinct. Knowledge pertains to an individual’s understanding of obesity, including its causes, health impacts, and management strategies [2, 4, 5]. Attitudes represent emotional responses toward adults living with obesity, influencing communication and clinical decisions [1, 4]. Beliefs reflect personal perceptions about obesity, shaped by views on responsibility and stigma [6, 7]. Although these constructs interact, it is essential to clearly define them to prevent conceptual overlap and misinterpretations in the development of an outcome measurement instrument (OMI) [8, 9].

In healthcare research, the KAB model is used to understand these constructs in clinical practice, including HCPs’ perspectives of adults living with obesity [9]. Evidence indicates that HCPs’ often have limited knowledge and negative attitudes towards adults living with obesity [6, 7, 10]. Such perceptions can negatively impact clinical decision‐making, patient engagement and overall quality of care, leading to diminished trust in healthcare services and poorer patient experiences [11, 12]. However, the accuracy and extent of this characterisation depend on the validity, reliability and conceptual strength of the OMI utilised in previous research within relevant healthcare populations [9, 13, 14]. Considering the recognised influence of weight stigma and HCPs’ beliefs on obesity management, there is a clear need for a robust, psychometrically sound OMI to accurately assess HCPs’ KAB. Given the complexity of the KAB model, a psychometrically sound OMI is essential; a poorly designed OMI can skew research findings and impede clinical practice and improvement [13].

Health constructs are inherently multidimensional and cannot be effectively captured through simplistic, unidimensional OMIs [8]. This is especially evident for obesity, a complex condition influenced by genetics, behaviour, socioeconomic status and environmental conditions [5, 15]. To accurately evaluate HCPs’ KAB about adult obesity, a comprehensive and valid OMI is required to represent their perspectives.

The Consensus‐based Standards for the Selection of Health Status Measurement Instruments (COSMIN) initiative is a standardised framework for selecting and evaluating healthcare measurement instruments, addressing issues such as inconsistent terminology and validation challenges [9]. By offering quality standards, COSMIN aims to ensure both measurement accuracy and reliability, and it has been widely embraced within healthcare research [9].

Research indicates that HCPs’ frequently possess limited knowledge and hold stigmatising attitudes towards patients with obesity, which negatively impacts the quality of care they provide [6, 7, 10, 11]. Therefore, ensuring the validity and reliability of the OMIs used in these studies is crucial for accurately identifying and addressing these challenges.

This research aims to identify and evaluate published OMIs that evaluate HCPs’ KAB about adult obesity, utilising the COSMIN framework. The goal is to critically appraise the psychometric robustness and applicability of existing OMIs within the HCP population.

## 2. Methods

### 2.1. Objectives

1. Identify published OMIs assessing HCPs’ KAB about adult obesity.
2. Critically evaluate the measurement properties and quality of evidence supporting these OMIs.

### 2.2. Protocol and Registration

This scoping review was not prospectively registered. It employed the COSMIN methodology for systematic reviews of patient‐reported outcome measures (PROMs) [16]. Additionally, it adhered to the five‐stage scoping review framework proposed by Arksey and O’Malley, incorporating Levac and Colquhoun’s refinements [17, 18]. The reporting was structured in accordance with the PRISMA‐ScR guidelines [19].

In this review, the term OMI is used to reflect HCPs’ reported use of measurement tools rather than a PROM. This terminology aligns with the COSMIN guidance.

### 2.3. Search Strategy

A systematic search was conducted across Embase, Medline (via EBSCO), Web of Science (core collection), PsycINFO, CINAHL (complete) and Google Scholar, encompassing publications from 1 January 1991 to 15 April 2023. PubMed was not searched separately due to substantial coverage overlap with Medline (via EBSCO). An updated search was completed from 15 April 2023 to 1 May 2025.

Search strategies, informed by COSMIN recommendations, targeted three primary components: (1) population (HCPs), (2) constructs of interest (KAB related to adult obesity) and (3) measurement properties (including development processes and psychometric qualities such as content validity, internal consistency and construct validity).

Search strategies were developed to identify OMIs that measure KAB. Specific terms related to each construct were used to address the variations in terminology and conceptual distinctions found in the literature. Full search strategies are detailed in Supporting Table 1.

### 2.4. Inclusion Criteria

Articles were included if they:

• Focused on OMIs assessing HCPs’ KAB about adult obesity.
• Targeted HCP populations.
• Described OMI development, psychometric evaluation or application.
• Were published post‐1991.
• Were available in full‐text format.
• Only English‐language full‐text articles were included.

### 2.5. Study Screening

Two reviewers independently conducted the literature screening and data extraction, with a third reviewer involved when consensus was needed. Article selection adhered to the COSMIN recommendations for evaluating measurement properties.

### 2.6. Data Extraction

Data extraction for Objective 1 focused on identifying published OMIs assessing HCPs’ KAB about adult obesity, including their development processes, target constructs, item domains and any psychometric evaluations reported.

For Objective 2, data extraction included methodological details relevant to psychometric evaluation, such as sample characteristics and validation procedures, to inform critical appraisal using the COSMIN checklist.

### 2.7. Quality Appraisal and Data Synthesis

Measurement properties were appraised using the COSMIN Risk of Bias checklist [9], evaluating:

• Content validity.
• Structural validity.
• Internal consistency.
• Reliability.
• Measurement error.
• Criterion validity.
• Construct validity.
• Cross‐cultural validity.
• Responsiveness.
• Overall risk of bias.

Each property was rated as ‘sufficient’ (+), ‘insufficient’ (−), ‘indeterminate’ (?), or ‘inconsistent’ (±). Emphasis was placed on content validity, the internal structure (including internal consistency and structural validity) and measurement reliability in line with COSMIN prioritisation principles.

### 2.8. Grading the Evidence

The strength of the evidence for each measurement property was then assessed using a modified GRADE approach, as recommended by the COSMIN methodology. This involved evaluating:

• Risk of Bias (based on COSMIN checklist ratings).
• Inconsistency.
• Indirectness.
• Imprecision
• Publication bias.

Evidence was graded as ‘High’, ‘Moderate’, ‘Low’ or ‘Very Low’.

### 2.9. Instrument Categorisation

OMIs were categorised according to COSMIN guidelines:

 Category A: Recommended—OMIs with sufficient content validity and at least low‐quality evidence for sufficient internal consistency.
 Category B: Potential to be recommended—OMIs requiring further validation.
 Category C: Not recommended—OMIs with high‐quality evidence of insufficient measurement properties.

## 3. Results

### 3.1. Search Results

A total of 9213 records were identified through database searches: 7670 from Search 1, 1543 from Search 2, and Google Scholar did not yield additional eligible studies. After excluding 3759 records due to duplicates and noneligible criteria (such as nonjournal articles and non‐English abstracts), 5454 records remained for abstract screening (4977 from Search 1 and 477 from Search 2).

Following the abstract screening process, 45 full‐text articles were identified and evaluated for eligibility. Among these, 41 articles were excluded, resulting in 4 articles being deemed appropriate for assessment under Objective 2, which aims to evaluate the methodological quality and measurement properties of the identified OMIs.

**FIGURE 1:**
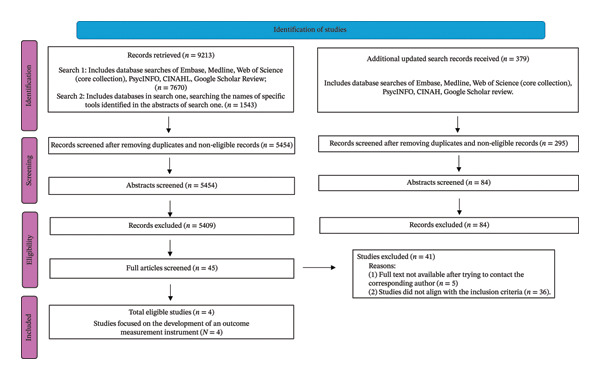
PRISMA flow diagram of scoping review.

An updated search across all databases conducted from 15 April 2023 to 1 May 2025 yielded an additional 379 records. After excluding 295 due to duplication and ineligibility, 84 records remained for abstract screening. However, no new articles met the criteria for full‐text review. Consequently, the final total number of eligible articles remained at four. See Figure 1 (PRISMA flow diagram) and Supporting Table 2 for database search results.

### 3.2. Study Characteristics and COSMIN Assessment

Supporting Table 2 summarises the key features of the included OMI studies. It provides details on the OMI name, developer, constructs, country and language, target population, mode of administration (e.g., self‐report), (sub) scales and item count, response options and scoring range.

The COSMIN Risk of Bias checklist assessment results are presented in Table 1.

**TABLE 1:** COSMIN Risk of Bias checklist assessment of outcome measurement instrument (OMI) development.

OMI	ORK ‐ 10	FPS	IAT	EFTB	OPS
Comprehensiveness	D	—	—	—	—
Comprehensibility	D	—	—	—	—
Cognitive interview sample	D	I	I	I	I
Concept elicitation	V	V	I	I	I
Representative sample	I	I	I	I	A
Context	V	V	V	V	V
Target population	V	V	V	V	I
Origin	V	D	D	D	D
Construct	V	V	V	V	I
Overall, COSMIN rating	I	I	I	I	I

*Note:* This table summarises the methodological quality of the development study for each included OMI, assessed using the COSMIN Risk of Bias checklist. The overall methodological quality rating was determined using the COSMIN worst score counts principle, whereby the lowest rating assigned across the relevant standards determines the overall rating. Ratings: V = very good; A = adequate; D = doubtful; I = inadequate; ‐ = not applicable/not assessed.

Abbreviations: EFTB = explicit fat–thin bias, FPS = Fat Phobia Scale, IAT = implicit association test, OMI = outcome measurement instrument, OPS = Obesity Perception Survey, ORK‐10 = Obesity Risk Knowledge Scale.

In the four studies analysed, none of the OMIs exclusively evaluated HCPs’ KAB in relation to adult obesity. Two OMIs assessed attitudinal biases or stigma towards individuals with obesity; one study focused on knowledge related to obesity and awareness of health risks, and another measured implicit bias among HCPs’.

All the studies assessed received an overall ‘inadequate’ rating for the development of OMIs. Key issues included the following.

In three of the four studies [20–23], the sample used during OMIs development was not representative of the target HCPs’ population.

The remaining study [23], while using an appropriate sample, did not clearly define the construct, it aimed to measure.

### 3.3. Application of the COSMIN Stop Rule

In accordance with COSMIN guidelines [9], OMIs that were rated ‘inadequate’ in the development phase should not undergo further psychometric property assessment. Given that all included OMIs failed to meet foundational development standards, further evaluation of measurement properties was not conducted.

## 4. Discussion

This scoping review identified four relevant articles focused on the development of OMIs. COSMIN appraisal indicated that the overall methodological quality of the OMIs’ development studies was inadequate.

Three of the four development studies demonstrated insufficient methodological rigour, particularly in recruiting samples representative of the HCP population [20–22]. One study, while including a representative sample, lacked a clear description of the construct being measured [23]. Although several OMIs were identified, there was a notable lack of HCP input during their development. This raises concerns about the acceptability and relevance of these OMIs for accurately capturing HCPs’ KAB about adult obesity.

Robust, population‐specific OMIs are essential to ensure that the perspectives of HCPs’ are accurately measured. The COSMIN framework emphasises the importance of selecting OMIs that are sensitive to both populations and context to enhance content validity [9]. Without such robust OMIs, understanding the complexities of HCP perspectives and identifying the barriers that influence their engagement with obesity management is significantly hindered [9, 12].

For instance, Wynn [24] utilised the Attitudes Towards Obese Persons (ATOP) and the Obesity Knowledge Scale (OKS) to evaluate obesity prejudice and knowledge among HCPs’. Their findings indicated no significant correlation between knowledge and prejudice levels, suggesting a disconnection between HCPs’ factual understanding and the biases they may hold. This observation supports the findings of the current review and underscores a broader issue: Existing OMIs fail to capture the complex, multifaceted nature of HCPs’ perspectives on adult obesity management. A recurring problem identified in the reviewed articles was the lack of clear definitions and distinctions between KAB. Given the intricate interplay among these constructs, which often influence one another [3, 4], a lack of conceptual clarity risks conflating them within OMI development. This confusion compromises both the interpretability and practical utility of OMI scores for the intended population.

A likely explanation for the methodological shortcomings of these OMIs is that they were developed prior to the introduction of the COSMIN checklist in 2010 [9]. As a result, these studies did not follow the structured processes recommended by the COSMIN framework, which is considered the best practice in OMI development. Similar findings have been reported in other COSMIN‐based reviews [25, 26], where OMIs were found to lack comprehensive development and validation processes, raising concerns about their quality and utility.

However, the majority of reviews have focused on the psychometric performance of weight stigma measures in the general population, rather than on the development quality and conceptual foundations of OMIs used with HCPs. For example, Papadopoulos et al. (2021) [14] assessed self‐reported weight stigma measurement instruments but did not examine whether these OMIs were specifically developed with input from HCPs’ or were intended to assess broader KAB about adults with obesity. In contrast, the current review advances the previous research by applying the COSMIN framework to the processes surrounding OMIs and specifically evaluating those used to gauge HCPs’ KAB about adult obesity. This highlights a significant gap in the availability of fit‐for‐purpose OMIs.

Obesity is a complex issue characterised by multiple influencing factors, and weight stigma remains an ongoing challenge across various populations. This underscores the importance of adopting measurement approaches that are both conceptually robust and attuned to the social and psychological contexts in which they operate [6, 27]. Applying the COSMIN framework shows that we cannot confidently assert that the current OMIs effectively and reliably capture HCPs’ KAB about adult obesity. This revelation underscores a significant gap and emphasises the need to develop psychometrically robust OMIs specifically designed for HCPs’, aligned with contemporary guidelines, COSMIN best practices and prior evidence highlighting weaknesses in healthcare OMI development [26].

The review also sought to facilitate comparisons among existing OMIs to support instrument selection. Limitations of this review include its restriction to English‐language publications, the potential omission of unpublished or the grey literature and reliance on published reports when applying COSMIN criteria. Older studies may not have detailed their OMI development processes sufficiently to meet contemporary methodological standards, potentially affecting the quality appraisal ratings. Additionally, due to the shortcomings in OMI development, content validity could not be confirmed, rendering further psychometric evaluations, such as GRADE assessments, inapplicable [9].

Without such OMIs, there remains a challenge in effectively evaluating HCP perspectives and in driving evidence‐informed interventions to improve obesity management. These findings underscore the need for new COSMIN‐aligned OMIs to assess HCPs’ KAB about adult obesity. Developing such high‐quality tools would enhance training, support stigma‐reducing initiatives, facilitate service evaluation and ultimately improve the standard of care in adult obesity management.

## 5. Conclusion

This scoping review identified only four published OMIs assessing HCPs’ KAB regarding adult obesity. Application of the COSMIN framework indicated that none demonstrated sufficient methodological quality or evidence of content validity. Future research should prioritise the development and validation of HCP‐specific OMIs that adhere to contemporary COSMIN standards to support obesity research, education, service evaluation and clinical practice.

## Author Contributions

Paul MacDonald conceived the study and was responsible for data collection, screening, data extraction, COSMIN appraisal and drafting the manuscript. Derek Peters, Helen Frank and Rebecca Stack contributed to the study design, provided methodological oversight, interpreted the findings and performed critical revisions of the manuscript.

## Funding

This study is part of a PhD, which is self‐funded by the author. Additional financial support to support tuition fees has been provided through charitable grants from the Chartered Society of Physiotherapy Charitable Trust and the Private Physiotherapy Educational Foundation (PPEF) for tuition fees. The authors have also received tuition fee support from Herefordshire and Worcestershire Health and Care NHS Trust.

## Disclosure

All authors approved the final version.

## Ethics Statement

In accordance with the University of Worcester Research Ethics Policy, ethical approval was not required for this scoping review, as it involved the analysis of previously published literature and did not include human participants, patient data or identifiable information.

## Conflicts of Interest

The authors declare no conflicts of interest.

## Supporting Information

Additional supporting information can be found online in the Supporting Information section.

## Supporting information


**Supporting Information 1** Supporting Table 1: Full systematic search strategies used across selected databases (Embase, Medline, Web of Science, PsycINFO, CINAHL and Google Scholar), including Boolean operators, search terms and database‐specific filters, to identify instruments measuring healthcare professionals’ knowledge, attitudes and beliefs (KAB) related to adult obesity.

**Supporting Information 2** Supporting Table 2: The database search summary outlines the key features of included studies on instruments, detailing the instrument name, developer, constructs, country and language, target population, mode of administration (e.g., self‐report), (sub) scales and item count, response options and scoring range.

## Data Availability

All data generated or analysed during this study are included within the published article and its supporting materials. No additional datasets were generated.
