# Supplementary material for: A Scoping Review of Outcome Measurement Instruments Assessing Healthcare Professionals’ Knowledge, Attitudes and Beliefs About Adult Obesity
Source: J Obes. 2026 Aug 13;2026:1363752. doi: 10.1155/jobe/1363752 (PMC13470226; doi:10.1155/jobe/1363752)
Supplement: Supplementary file 2 [file JOBE-2026-1363752-s001.zip › Supplementary Table 1.docx]

**Literature Search**

Searches covered publications from 1^st^ January 1991 to 15^th^ April 2023 and were updated to include studies published up to 1^st^ May 2025. No additional eligible studies were identified in the updated search.

**Health Professional-related Databases:**

- Embase
- Medline
- Web of Science (core collection)
- PsycINFO
- CINAHL (complete)
- Google Scholar

**Search Boolean:**

Attitudes and Beliefs Outcome Measures

1. obesity or overweight or fat or obese or unhealthy weight or high bmi
2. attitudes or perceptions or opinions or thoughts or feelings or beliefs or views or attitudes towards
3. adults or adult or aged or elderly or middle aged or older person or young adult
4. outcome measure or measure or instrument or questionnaire or assessment or evaluation or scale or test or tool
5. clinicians or health professionals or nurses or physicians or doctors or physiotherapists or physical therapists or allied health or therapist
6. psychometrics or validity or reliability or psychometric properties

knowledge Outcome Measures

1. obesity or overweight or fat or obese or unhealthy weight or high bmi
2. knowledge or education or understanding or awareness or training or skill
3. adults or adult or aged or elderly or middle aged or older person or young adult
4. outcome measure or measure or instrument or questionnaire or assessment or evaluation or scale or test or tool
5. clinicians or health professionals or nurses or physicians or doctors or physiotherapists or physical therapists or allied health or therapist
6. psychometrics or validity or reliability or psychometric properties

- **Search 1 Table:**

| Database Searched | Search Terms Used | Number of Results | Comments of Results | Date of Search |
| --- | --- | --- | --- | --- |
| **CINAHL via EBSCO**  **Attitudes and Beliefs**  **Search 1** | obesity or overweight or fat or obese or unhealthy weight or high bmi  AND attitudes or perceptions or opinions or thoughts or feelings or beliefs or views or attitudes towards  AND adults or adult or aged or elderly or middle aged or older person or young adult  AND outcome measure or measure or instrument or questionnaire or assessment or evaluation or scale or test or tool | 5,984 | Too large a search field.  Need to be more specific focus on use of outcome measures used by HCP’s. | 06/02/2023 |
| **CINAHL via EBSCO**  **Attitudes and Beliefs**  **Search 2** | obesity or overweight or fat or obese or unhealthy weight or high bmi  AND attitudes or perceptions or opinions or thoughts or feelings or beliefs or views or attitudes towards  AND adults or adult or aged or elderly or middle aged or older person or young adult  AND outcome measure or measure or instrument or questionnaire or assessment or evaluation or scale or test or tool  AND clinicians or health professionals or nurses or physicians or doctors or physiotherapists or physical therapists or allied health or therapist | 848 | Moderate number of relevant articles for full abstract assessment.  Import to Zotero and for removal of duplicates and those that do not fulfil inclusion criteria.  Conduct more specific search of psychometric properties to focus search field – see search 3 | 06/02/2023 |
| **CINAHL via EBSCO**  **Attitudes and Beliefs**  **Search 3** | obesity or overweight or fat or obese or unhealthy weight or high bmi  AND attitudes or perceptions or opinions or thoughts or feelings or beliefs or views or attitudes towards  AND adults or adult or aged or elderly or middle aged or older person or young adult  AND outcome measure or measure or instrument or questionnaire or assessment or evaluation or scale or test or tool  AND clinicians or health professionals or nurses or physicians or doctors or physiotherapists or physical therapists or allied health or therapist  AND psychometrics or validity or reliability or psychometric properties | 60 | More relevant articles for full abstract assessment.  Import to Zotero.    Note all articles are duplicated from Search 2. | 06/02/2023 |
|  |  |  |  |  |
| **CINAHL via EBSCO – Knowledge**  **Search 1** | obesity or overweight or fat or obese or unhealthy weight or high bmi  AND knowledge or education or understanding or awareness or training or skill  AND adults or adult or aged or elderly or middle aged or older person or young adult  AND outcome measure or measure or instrument or questionnaire or assessment or evaluation or scale or test or tool | 13,466 | Too large a search field.  Need to be more specific focus on use of outcome measures used by HCP’s. | 06/02/2023 |
| **CINAHL via EBSCO – Knowledge**  **Search 2** | obesity or overweight or fat or obese or unhealthy weight or high bmi  AND knowledge or education or understanding or awareness or training or skill  AND adults or adult or aged or elderly or middle aged or older person or young adult  AND outcome measure or measure or instrument or questionnaire or assessment or evaluation or scale or test or tool  AND clinicians or health professionals or nurses or physicians or doctors or physiotherapists or physical therapists or allied health or therapist | 1,456 | Moderate number of relevant articles for full abstract assessment.  Import to Zotero and for removal of duplicates and those that do not fulfil inclusion criteria.  Conduct more specific search of psychometric properties to focus search field – see search 3 | 06/02/2023 |
| **CINAHL via EBSCO – Knowledge**  **Search 3** | obesity or overweight or fat or obese or unhealthy weight or high bmi  AND knowledge or education or understanding or awareness or training or skill  AND adults or adult or aged or elderly or middle aged or older person or young adult  AND outcome measure or measure or instrument or questionnaire or assessment or evaluation or scale or test or tool  AND clinicians or health professionals or nurses or physicians or doctors or physiotherapists or physical therapists or allied health or therapist  AND psychometrics or validity or reliability or psychometric properties | 81 | More relevant articles for full abstract assessment.  Import to Zotero.    Note all articles are duplicated from Search 2. | 06/02/2023 |
|  |  |  |  |  |
| **MEDLINE via EBSCO**  **Attitudes and Beliefs**  **Search 1** | obesity or overweight or fat or obese or unhealthy weight or high bmi  AND attitudes or perceptions or opinions or thoughts or feelings or beliefs or views or attitudes towards  AND adults or adult or aged or elderly or middle aged or older person or young adult  AND outcome measure or measure or instrument or questionnaire or assessment or evaluation or scale or test or tool | 11,531 | Too large a search field.  Need to be more specific focus on use of outcome measures used by HCP’s. | 13/02/2023 |
| **MEDLINE via EBSCO**  **Attitudes and Beliefs**  **Search 2** | obesity or overweight or fat or obese or unhealthy weight or high bmi  AND attitudes or perceptions or opinions or thoughts or feelings or beliefs or views or attitudes towards  AND adults or adult or aged or elderly or middle aged or older person or young adult  AND outcome measure or measure or instrument or questionnaire or assessment or evaluation or scale or test or tool  AND clinicians or health professionals or nurses or physicians or doctors or physiotherapists or physical therapists or allied health or therapist | 1,554 | Moderate number of relevant articles for full abstract assessment.  Import to Zotero and for removal of duplicates and those that do not fulfil inclusion criteria.  Conduct more specific search of psychometric properties to focus search field – see search 3 | 13/02/2023 |
| **MEDLINE via EBSCO**  **Attitudes and Beliefs**  **Search 3** | obesity or overweight or fat or obese or unhealthy weight or high bmi  AND attitudes or perceptions or opinions or thoughts or feelings or beliefs or views or attitudes towards  AND adults or adult or aged or elderly or middle aged or older person or young adult  AND outcome measure or measure or instrument or questionnaire or assessment or evaluation or scale or test or tool  AND clinicians or health professionals or nurses or physicians or doctors or physiotherapists or physical therapists or allied health or therapist  AND psychometrics or validity or reliability or psychometric properties | 55 | More relevant articles for full abstract assessment.  Import to Zotero.    Note all articles are duplicated from Search 2. | 13/02/2023 |
|  |  |  |  |  |
| **Medline via EBSCO – Knowledge**  **Search 1** | obesity or overweight or fat or obese or unhealthy weight or high bmi  AND knowledge or education or understanding or awareness or training or skill  AND adults or adult or aged or elderly or middle aged or older person or young adult  AND outcome measure or measure or instrument or questionnaire or assessment or evaluation or scale or test or tool | 29,581 | Too large a search field.  Need to be more specific focus on use of outcome measures used by HCP’s. | 13/02/2023 |
| **Medline via EBSCO – Knowledge**  **Search 2** | obesity or overweight or fat or obese or unhealthy weight or high bmi  AND knowledge or education or understanding or awareness or training or skill  AND adults or adult or aged or elderly or middle aged or older person or young adult  AND outcome measure or measure or instrument or questionnaire or assessment or evaluation or scale or test or tool  AND clinicians or health professionals or nurses or physicians or doctors or physiotherapists or physical therapists or allied health or therapist | 3,063 | Too large a search field.  Need to be more specific focus articles for full abstract assessment.  Conduct more specific search of psychometric properties to focus search field – see search 3 | 13/02/2023 |
| **Medline via EBSCO – Knowledge**  **Search 3** | obesity or overweight or fat or obese or unhealthy weight or high bmi  AND knowledge or education or understanding or awareness or training or skill  AND adults or adult or aged or elderly or middle aged or older person or young adult  AND outcome measure or measure or instrument or questionnaire or assessment or evaluation or scale or test or tool  AND clinicians or health professionals or nurses or physicians or doctors or physiotherapists or physical therapists or allied health or therapist  AND psychometrics or validity or reliability or psychometric properties | 96 | More relevant articles for full abstract assessment.  Import to Zotero.    Note all articles are duplicated from Search 2. | 13/02/2023 |
|  |  |  |  |  |
| **APA PsycINFO via EBSCO**  **Attitudes and Beliefs**  **Search 1** | obesity or overweight or fat or obese or unhealthy weight or high bmi  AND attitudes or perceptions or opinions or thoughts or feelings or beliefs or views or attitudes towards  AND adults or adult or aged or elderly or middle aged or older person or young adult  AND outcome measure or measure or instrument or questionnaire or assessment or evaluation or scale or test or tool | 5,135 | Too large a search field.  Need to be more specific focus on use of outcome measures used by HCP’s. | 13/02/2023 |
| **APA PsycINFO via EBSCO**  **Attitudes and Beliefs**  **Search 2** | obesity or overweight or fat or obese or unhealthy weight or high bmi  AND attitudes or perceptions or opinions or thoughts or feelings or beliefs or views or attitudes towards  AND adults or adult or aged or elderly or middle aged or older person or young adult  AND outcome measure or measure or instrument or questionnaire or assessment or evaluation or scale or test or tool | 523 | Moderate number of relevant articles for full abstract assessment.  Import to Zotero and for removal of duplicates and those that do not fulfil inclusion criteria.  Conduct more specific search of psychometric properties to focus search field – see search 3 |  |
| **APA PsycINFO via EBSCO**  **Attitudes and Beliefs**  **Search 3** | obesity or overweight or fat or obese or unhealthy weight or high bmi  AND attitudes or perceptions or opinions or thoughts or feelings or beliefs or views or attitudes towards  AND adults or adult or aged or elderly or middle aged or older person or young adult  AND outcome measure or measure or instrument or questionnaire or assessment or evaluation or scale or test or tool  AND clinicians or health professionals or nurses or physicians or doctors or physiotherapists or physical therapists or allied health or therapist  AND psychometrics or validity or reliability or psychometric properties | 55 | More relevant relevant articles for full abstract assessment.  Import to Zotero.    Note all articles are duplicated from Search 2. | 13/02/2023 |
|  |  |  |  |  |
| **APA PsycINFO via EBSCO – Knowledge**  **Search 1** | obesity or overweight or fat or obese or unhealthy weight or high bmi  AND knowledge or education or understanding or awareness or training or skill  AND adults or adult or aged or elderly or middle aged or older person or young adult  AND outcome measure or measure or instrument or questionnaire or assessment or evaluation or scale or test or tool | 6,810 | Too large a search field.  Need to be more specific focus on use of outcome measures used by HCP’s. | 13/02/2023 |
| **APA PsycINFO via EBSCO – Knowledge**  **Search 2** | obesity or overweight or fat or obese or unhealthy weight or high bmi  AND knowledge or education or understanding or awareness or training or skill  AND adults or adult or aged or elderly or middle aged or older person or young adult  AND outcome measure or measure or instrument or questionnaire or assessment or evaluation or scale or test or tool  AND clinicians or health professionals or nurses or physicians or doctors or physiotherapists or physical therapists or allied health or therapist | 708 | Moderate number of relevant articles for full abstract assessment.  Import to Zotero and for removal of duplicates and those that do not fulfil inclusion criteria.  Conduct more specific search of psychometric properties to focus search field – see search 3 | 20/02/2023 |
| **APA PsycINFO via EBSCO – Knowledge**  **Search 3** | obesity or overweight or fat or obese or unhealthy weight or high bmi  AND knowledge or education or understanding or awareness or training or skill  AND adults or adult or aged or elderly or middle aged or older person or young adult  AND outcome measure or measure or instrument or questionnaire or assessment or evaluation or scale or test or tool  AND clinicians or health professionals or nurses or physicians or doctors or physiotherapists or physical therapists or allied health or therapist  AND psychometrics or validity or reliability or psychometric properties | 31 | More relevant articles for full abstract assessment.  Import to Zotero.    Note all articles are duplicated from Search 2. | 20/02/2023 |
|  |  |  |  |  |
| **EMBASE**  **Attitudes and Beliefs**  **Search 1** | obesity or overweight or fat or obese or unhealthy weight or high bmi  AND attitudes or perceptions or opinions or thoughts or feelings or beliefs or views or attitudes towards  AND adults or adult or aged or elderly or middle aged or older person or young adult  AND outcome measure or measure or instrument or questionnaire or assessment or evaluation or scale or test or tool | 7947 | Too large a search field.  Need to be more specific focus on use of outcome measures used by HCP’s. | 20/02/2023 |
| **EMBASE**  **Attitudes and Beliefs**  **Search 2** | obesity or overweight or fat or obese or unhealthy weight or high bmi  AND attitudes or perceptions or opinions or thoughts or feelings or beliefs or views or attitudes towards  AND adults or adult or aged or elderly or middle aged or older person or young adult  AND outcome measure or measure or instrument or questionnaire or assessment or evaluation or scale or test or tool  AND clinicians or health professionals or nurses or physicians or doctors or physiotherapists or physical therapists or allied health or therapist | 998 | Low number of relevant articles for full abstract assessment.  Need to be more specific focus articles for full abstract assessment.  Conduct more specific search of psychometric properties to focus search field – see search 3 | 20/02/2023 |
| **EMBASE**  **Attitudes and Beliefs**  **Search 3** | obesity or overweight or fat or obese or unhealthy weight or high bmi  AND attitudes or perceptions or opinions or thoughts or feelings or beliefs or views or attitudes towards  AND adults or adult or aged or elderly or middle aged or older person or young adult  AND outcome measure or measure or instrument or questionnaire or assessment or evaluation or scale or test or tool  AND clinicians or health professionals or nurses or physicians or doctors or physiotherapists or physical therapists or allied health or therapist  AND psychometrics or validity or reliability or psychometric properties | 42 | More relevant articles for full abstract assessment.  Import to Zotero.    Note all articles are duplicated from Search 2. | 20/02/2023 |
|  |  |  |  |  |
| **EMBASE**  **Knowledge**  **Search 1** | obesity or overweight or fat or obese or unhealthy weight or high bmi AND knowledge or education or understanding or awareness or training or skill AND adults or adult or aged or elderly or middle aged or older person or young adult AND outcome measure or measure or instrument or questionnaire or assessment or evaluation or scale or test or tool | 52488 | Too large a search field.  Need to be more specific focus on use of outcome measures used by HCP’s. | 27/02/2023 |
| **EMBASE**  **Knowledge**  **Search 2** | obesity or overweight or fat or obese or unhealthy weight or high bmi AND knowledge or education or understanding or awareness or training or skill AND adults or adult or aged or elderly or middle aged or older person or young adult AND outcome measure or measure or instrument or questionnaire or assessment or evaluation or scale or test or tool AND clinicians or health professionals or nurses or physicians or doctors or physiotherapists or physical therapists or allied health or therapist | 4206 | Too large a search field.  Need to be more specific focus articles for full abstract assessment.  Conduct more specific search of psychometric properties to focus search field – see search 3 | 27/02/2023 |
| **EMBASE**  **Knowledge**  **Search 3** | obesity or overweight or fat or obese or unhealthy weight or high bmi AND knowledge or education or understanding or awareness or training or skill  adults or adult or aged or elderly or middle aged or older person or young adult AND outcome measure or measure or instrument or questionnaire or assessment or evaluation or scale or test or tool AND clinicians or health professionals or nurses or physicians or doctors or physiotherapists or physical therapists or allied health or therapist AND psychometrics or validity or reliability or psychometric properties | 137 | More relevant articles for full abstract assessment.  Import to Zotero.    Note all articles are duplicated from Search 2. | 27/02/2023 |
|  |  |  |  |  |
| **WEB OF SCIENCE**  **Attitudes and Beliefs**  **Search 1** | obesity or overweight or fat or obese or unhealthy weight or high bmi  AND attitudes or perceptions or opinions or thoughts or feelings or beliefs or views or attitudes towards  AND adults or adult or aged or elderly or middle aged or older person or young adult  AND outcome measure or measure or instrument or questionnaire or assessment or evaluation or scale or test or tool | 17563 | Too large a search field.  Need to be more specific focus on use of outcome measures used by HCP’s. | 27/02/2023 |
| **WEB OF SCIENCE**  **Attitudes and Beliefs**  **Search 2** | obesity or overweight or fat or obese or unhealthy weight or high bmi  AND attitudes or perceptions or opinions or thoughts or feelings or beliefs or views or attitudes towards  AND adults or adult or aged or elderly or middle aged or older person or young adult  AND outcome measure or measure or instrument or questionnaire or assessment or evaluation or scale or test or tool  AND clinicians or health professionals or nurses or physicians or doctors or physiotherapists or physical therapists or allied health or therapist | 3038 | Too large a search field.  Need to be more specific focus articles for full abstract assessment.  Conduct more specific search of psychometric properties to focus search field – see search 3 | 27/02/2023 |
| **WEB OF SCIENCE**  **Attitudes and Beliefs**  **Search 3** | obesity or overweight or fat or obese or unhealthy weight or high bmi  AND attitudes or perceptions or opinions or thoughts or feelings or beliefs or views or attitudes towards  AND adults or adult or aged or elderly or middle aged or older person or young adult  AND outcome measure or measure or instrument or questionnaire or assessment or evaluation or scale or test or tool  AND clinicians or health professionals or nurses or physicians or doctors or physiotherapists or physical therapists or allied health or therapist  AND psychometrics or validity or reliability or psychometric properties | 279 | Good / relevant articles for full abstract assessment. – will import to Zotero and remove duplicates and those that do not fulfil inclusion criteria | 27/02/2023 |
|  |  |  |  |  |
| **WEB OF SCIENCE**  **Knowledge**  **Search 1** | obesity or overweight or fat or obese or unhealthy weight or high bmi AND knowledge or education or understanding or awareness or training or skill AND adults or adult or aged or elderly or middle aged or older person or young adult AND outcome measure or measure or instrument or questionnaire or assessment or evaluation or scale or test or tool | 55203 | Too large a search field.  Need to be more specific focus on use of outcome measures used by HCP’s. | 06/03/2023 |
| **WEB OF SCIENCE**  **Knowledge**  **Search 2** | obesity or overweight or fat or obese or unhealthy weight or high bmi AND knowledge or education or understanding or awareness or training or skill AND adults or adult or aged or elderly or middle aged or older person or young adult AND outcome measure or measure or instrument or questionnaire or assessment or evaluation or scale or test or tool AND clinicians or health professionals or nurses or physicians or doctors or physiotherapists or physical therapists or allied health or therapist | 11851 | Too large a search field.  Need to be more specific focus articles for full abstract assessment.  Conduct more specific search of psychometric properties to focus search field – see search 3 | 06/03/2023 |
| **WEB OF SCIENCE**  **Knowledge**  **Search 3** | obesity or overweight or fat or obese or unhealthy weight or high bmi AND knowledge or education or understanding or awareness or training or skill AND adults or adult or aged or elderly or middle aged or older person or young adult AND outcome measure or measure or instrument or questionnaire or assessment or evaluation or scale or test or tool AND clinicians or health professionals or nurses or physicians or doctors or physiotherapists or physical therapists or allied health or therapist AND psychometrics or validity or reliability or psychometric properties | 1089 | More relevant articles for full abstract assessment.  Import to Zotero.    Note all articles are duplicated from Search 2. | 06/03/2023 |

- **Total Number of articles Uploaded to Zotero = 7,670**
- **Total number of removed duplications, non-journal articles (thesis, guidelines, book chapters) and non-English abstracts = 2693**
- **Total Number of articles for abstract review = 4977**

**Abstract Review:** 06/03/2023 - 26/03/2023

**Selection of relevant studies for full article review as per inclusion/ exclusion criteria below:**

**Inclusion Criteria**

- Timeframe – January 1^st^ 1991 till present
- original research studies published in peer-reviewed journals;
- Include at least one instrument for measuring knowledge, attitudes and beliefs of adult obesity healthcare professionals (ie, literature describing the development of the instrument; literature describing the psychometric testing of the instrument; and/or literature in which the instrument was used to measure the knowledge, attitudes and beliefs of adult obesity of healthcare professionals)
- have full text availability

**Exclusion Criteria**

- Non-English language papers
- Where full text articles could not be accessed
- OM(s) not specifically developed for measuring the knowledge, attitudes and beliefs of HCPs about adult obesity.
- OM(s) not published in peer reviewed journals.

**Total papers identified for full review = 29**

- **WENT BACK THROUGH REJECTIONS TO DOUBLE CHECK – NO FURTHER ABSTRACTS IDENTIFIED -** 27/03/2023

**Common Themes for exclusion –**

- **no clear/ specific outcome measurement used/ developed to assess attitudes, beliefs, or knowledge of obesity.**
- **non-HPC populations – general population or university students majority of papers**

**Second Search Strategy:**

***Identification of relevant measurement tools:*** To ensure a further review of the literature and that the information regarding development and testing of each of the identified tools was comprehensive, a further search of the online databases was carried out using the name of each of the specific tools of the previously identified abstracts.

**Outcome Measures identified in selected abstracts:**

1. Implicit Anti Fat Bias
2. Explicit Anti Fat Bias
3. Fat Phobia Scale (FPS)
4. Anti Fat Attitudes Questionnaire (AFA)
5. Beliefs About Obese People (BAOP)
6. Attitudes Towards Obese Persons Scale (ATOP)
7. Obesity Risk Knowledge Scale (ORK-10)
8. Obesity Perception Survey (OPS)

- **Search 2 Table:**

| Database Searched | Search Terms Used | Number of Results | Comments of Results | Date of Search |
| --- | --- | --- | --- | --- |
| **CINAHL/ MEDLINE/ APA Psych INFO via EBSCO** | Implicit Anti-Fat Bias | 50 | relevant articles for full abstract assessment.  Import to Zotero. | 03/04/2023 |
| **CINAHL/ MEDLINE/ APA Psych INFO via EBSCO** | Explicit Anti Fat Bias | 33 | relevant articles for full abstract assessment.  Import to Zotero. | 03/04/2023 |
| **CINAHL/ MEDLINE/ APA Psych INFO via EBSCO** | Fat Phobia Scale OR FPS | 9,931 | Too large a search field - need to be more specific focus on use of outcome measure | 03/04/2023 |
| **CINAHL/ MEDLINE/ APA Psych INFO via EBSCO** | Fat Phobia Scale | 128 | relevant articles for full abstract assessment.  Import to Zotero. | 03/04/2023 |
| **CINAHL/ MEDLINE/ APA Psych INFO via EBSCO** | Anti Fat Attitudes Questionnaire OR AFA | 2,006 | Too large a search field - need to be more specific focus on use of outcome measure | 03/04/2023 |
| **CINAHL/ MEDLINE/ APA Psych INFO via EBSCO** | Anti Fat Attitudes Questionnaire | 56 | relevant articles for full abstract assessment.  Import to Zotero. | 03/04/2023 |
| **CINAHL/ MEDLINE/ APA Psych INFO via EBSCO** | Beliefs About Obese People OR BAOP | 75 | relevant articles for full abstract assessment.  Import to Zotero. | 03/04/2023 |
| **CINAHL/ MEDLINE/ APA Psych INFO via EBSCO** | Attitudes towards obese persons OR ATOP | 2,202 | Too large a search field - need to be more specific focus on use of outcome measure | 03/04/2023 |
| **CINAHL/ MEDLINE/ APA Psych INFO via EBSCO** | Attitudes towards obese persons | 153 | relevant articles for full abstract assessment.  Import to Zotero. | 03/04/2023 |
| **CINAHL/ MEDLINE/ APA Psych INFO via EBSCO** | Obesity Risk Knowledge OR ORK-10 | 219 | relevant articles for full abstract assessment.  Import to Zotero. | 03/04/2023 |
| **CINAHL/ MEDLINE/ APA Psych INFO via EBSCO** | Obesity Prejudice Scale OR OPS | 5,772 | Too large a search field - need to be more specific focus on use of outcome measure | 03/04/2023 |
| **CINAHL/ MEDLINE/ APA Psych INFO via EBSCO** | Obesity Prejudice Scale | 5 | relevant articles for full abstract assessment.  Import to Zotero. | 03/04/2023 |
|  |  |  |  |  |
| **EMBASE** | Implicit Anti Fat Bias | 17 | relevant articles for full abstract assessment.  Import to Zotero. | 10/04/2023 |
| **EMBASE** | Explicit Anti Fat Bias | 10 | relevant articles for full abstract assessment.  Import to Zotero. | 10/04/2023 |
| **EMBASE** | Fat Phobia Scale OR FPS | 7908 | Too large a search field - need to be more specific focus on use of outcome measure | 10/04/2023 |
| **EMBASE** | Fat Phobia Scale | 73 | relevant articles for full abstract assessment.  Import to Zotero. | 10/04/2023 |
| **EMBASE** | Anti Fat Attitudes Questionnaire OR AFA | 1492 | Too large a search field - need to be more specific focus on use of outcome measure | 10/04/2023 |
| **EMBASE** | Anti Fat Attitudes Questionnaire | 16 | relevant articles for full abstract assessment.  Import to Zotero. | 10/04/2023 |
| **EMBASE** | Beliefs About Obese People OR BAOP | 40 | relevant articles for full abstract assessment.  Import to Zotero. | 10/04/2023 |
| **EMBASE** | Attitudes towards obese persons OR ATOP | 1628 | Too large a search field - need to be more specific focus on use of outcome measure | 10/04/2023 |
| **EMBASE** | Attitudes towards obese persons | 15 | relevant articles for full abstract assessment.  Import to Zotero. | 10/04/2023 |
| **EMBASE** | Obesity Risk Knowledge OR ORK-10 | 17 | Initially 20 - removal of duplicates and non-journals = 17 | 10/04/2023 |
| **EMBASE** | Obesity Prejudice Scale OR OPS | 5728 | Too large a search field - need to be more specific focus on use of outcome measure | 10/04/2023 |
| **EMBASE** | Obesity Prejudice Scale OR OPS | 5 | relevant articles for full abstract assessment.  Import to Zotero. | 10/04/2023 |
|  |  |  |  |  |
| **WEB OF SCIENCE** | Implicit Anti Fat Bias | 128 | relevant articles for full abstract assessment.  Import to Zotero. | 10/04/2023 |
| **WEB OF SCIENCE** | Explicit Anti Fat Bias | 87 | relevant articles for full abstract assessment.  Import to Zotero. | 10/04/2023 |
| **WEB OF SCIENCE** | Fat Phobia Scale OR FPS | 17211 | To large a search field - need to be more specific focus on use of outcome measures | 10/04/2023 |
| **WEB OF SCIENCE** | Fat Phobia Scale | 69 | relevant articles for full abstract assessment.  Import to Zotero. | 10/04/2023 |
| **WEB OF SCIENCE** | Anti Fat Attitudes Questionnaire OR AFA | 7804 | To large a search field - need to be more specific focus on use of outcome measures | 10/04/2023 |
| **WEB OF SCIENCE** | Anti Fat Attitudes Questionnaire | 65 | relevant articles for full abstract assessment.  Import to Zotero. | 10/04/2023 |
| **WEB OF SCIENCE** | Beliefs About Obese People OR BAOP | 171 | relevant articles for full abstract assessment.  Import to Zotero. | 10/04/2023 |
| **WEB OF SCIENCE** | Attitudes towards obese persons OR ATOP | 7703 | To large a search field - need to be more specific focus on use of outcome measures | 10/04/2023 |
| **WEB OF SCIENCE** | Attitudes towards obese persons OR ATOP | 7703 | To large a search field - need to be more specific focus on use of outcome measures | 10/04/2023 |
| **WEB OF SCIENCE** | Attitudes towards obese persons | 44 | relevant articles for full abstract assessment.  Import to Zotero. | 10/04/2023 |
| **WEB OF SCIENCE** | Obesity Risk Knowledge OR ORK-10 | 6944 | To large a search field - need to be more specific focus on use of outcome measures | 10/04/2023 |
| **WEB OF SCIENCE** | Obesity Risk Knowledge | 6944 | To large a search field - need to be more specific focus on use of outcome measures | 10/04/2023 |
| **WEB OF SCIENCE** | ORK-10 | 9 | relevant articles for full abstract assessment.  Import to Zotero. | 10/04/2023 |
| **WEB OF SCIENCE** | Obesity Prejudice Scale OR OPS | 9707 | To large a search field - need to be more specific focus on use of outcome measures | 10/04/2023 |
| **WEB OF SCIENCE** | Obesity Prejudice Scale | 75 | relevant articles for full abstract assessment.  Import to Zotero. | 10/04/2023 |

**11/04/2023**

- Total Number of Search items imported in to Zotero = 1543
- Total Number of Duplicates and non-journal articles removed = 1066
- Total Abstracts for review = 477

Total papers identified in Search 2 for full review that weren’t identified in Search 1 = 9

**WENT BACK THROUGH REJECTIONS TO DOUBLE CHECK – NO FURTHER ABSTRACTS IDENTIFIED.** – 13/04/2023

**Common Themes for exclusion –**

- no clear/ specific outcome measurement used/ developed to assess attitudes, beliefs, or knowledge of obesity.
- non-HPC populations – general population or university students

**Total number of papers identified in search 1 and 2 for full article review = 45**

**Google Scholar Review: 15/04/2023**

- Top 100 results were scanned for relevance utilising Boolean search strategy and use of named outcome measures – no additional research articles were identified.

**Full Article Review: 17/05/2023 -**

**Papers Identified for full article review: 45**

1. Akman M., Kivrakoglu E., Cifcili S., and Unalan P.C. ‘Weight Bias among Primary Care Health Professionals: Personal Attitude Matters’. *Obesity and Metabolism* 6, no. 2–3 (2010): 63–68.
2. ALLISON, D., BASILE, V., & YUKER, H. (1991). THE MEASUREMENT OF ATTITUDES TOWARD AND BELIEFS ABOUT OBESE PERSONS. INTERNATIONAL JOURNAL OF EATING DISORDERS, 10(5), 599–607.
3. Bacon, J., Scheltema, K., & Robinson, B. (2001). Fat phobia scale revisited: The short form. *INTERNATIONAL JOURNAL OF OBESITY*, *25*(2), 252–257. <https://doi.org/10.1038/sj.ijo.0801537>
4. Bøker Lund, Thomas, John Brodersen, and Peter Sandøe. ‘A Study of Anti-Fat Bias among Danish General Practitioners and Whether This Bias and General Practitioners’ Lifestyle Can Affect Treatment of Tension Headache in Patients with Obesity.’ *Obesity Facts* 11, no. 6 (2018): 501–13. <https://doi.org/10.1159/000493373>.
5. Cain, P., Donaghue, N., & Ditchburn, G. (2022). Development and validation of the Fat Attitudes Assessment Toolkit (FAAT): A multidimensional nonstigmatizing measure of contemporary attitudes toward fatness and fat people. *JOURNAL OF APPLIED SOCIAL PSYCHOLOGY*, *52*(12), 1121–1145. <https://doi.org/10.1111/jasp.12882>
6. CRANDALL, C. (1994). PREJUDICE AGAINST FAT PEOPLE - IDEOLOGY AND SELF-INTEREST. *JOURNAL OF PERSONALITY AND SOCIAL PSYCHOLOGY*, *66*(5), 882–894. <https://doi.org/10.1037/0022-3514.66.5.882>
7. Da Q Zhu, Ian J. Norman, and Alison E. While. ‘Nurses’ Self-Efficacy and Practices Relating to Weight Management of Adult Patients: A Path Analysis.’ *International Journal of Behavioral Nutrition & Physical Activity* 10 (January 2013): 131–53. <https://doi.org/10.1186/1479-5868-10-131>.
8. Darling, Rosa, and A. Serdar Atav. ‘Attitudes toward Obese People: A Comparative Study of Nursing, Education, and Social Work Students’. *Journal of Professional Nursing* 35, no. 2 (March 2019): 138–46. <https://doi.org/10.1016/j.profnurs.2018.07.009>.
9. Diversi, T M, R Hughes, and K J Burke. ‘The Prevalence and Practice Impact of Weight Bias amongst Australian Dietitians.’ *Obesity Science & Practice* 2, no. 4 (December 2016): 456–65. <https://doi.org/10.1002/osp4.83>.
10. Elboim-Gabyzon, M, K Attar, and S Peleg. ‘Weight Stigmatization among Physical Therapy Students and Registered Physical Therapists’. *OBESITY FACTS* 13, no. 2 (May 2020): 104–16. <https://doi.org/10.1159/000504809>.
11. Forhan M., Torti J., and Jones A. ‘Weight Bias among Healthcare Providers: Quality of Life Implications for Patients with Obesity’. *Quality of Life Research*, 22nd Annual Conference of the International Society for Quality of Life Research, ISOQOL. Vancouver, BC Canada., 24, no. 1 Supplement 1 (2015): 183. <https://doi.org/10.1007/s11136-015-1078-4>.
12. Gujral, Harpreet, Christine Tea, and Michael Sheridan. ‘Evaluation of Nurse’s Attitudes toward Adult Patients of Size.’ *Surgery for Obesity and Related Diseases : Official Journal of the American Society for Bariatric Surgery* 7, no. 4 (July 2011): 536–40. <https://doi.org/10.1016/j.soard.2011.03.008>.
13. Jones C.A. and Forhan M. ‘Addressing Weight Bias and Stigma of Obesity amongst Physiotherapists’. *Physiotherapy Theory and Practice*, no. (Jones, Forhan) Faculty of Rehabilitation Medicine, University of Alberta, 2-50 Corbett Hall, Edmonton, AB, Canada (2019): 1–9. <https://doi.org/10.1080/09593985.2019.1648623>.
14. Khandalavala B.N., Rojanala A., Geske J.A., Koran-Scholl J.B., and Guck T.P. ‘Obesity Bias in Primary Care Providers’. *Family Medicine* 46, no. 7 (2014): 532–35.
15. Lewis, R., Cash, T., Jacobi, L., & BubbLewis, C. (1997). Prejudice toward fat people: The development and validation of the antifat attitudes test. *OBESITY RESEARCH*, *5*(4), 297–307. <https://doi.org/10.1002/j.1550-8528.1997.tb00555.x>
16. MacLaughlin B.D. and Huber J.W. ‘Attitudes to Overweight and Obesity among Healthcare Professionals Working within Diabetes Care’. *Diabetic Medicine*, Diabetes UK Annual Professional Conference. Liverpool United Kingdom., 27, no. 2 SUPPL. 1 (2010): 108. <https://doi.org/10.1111/j.1464-5491.2009.02936.x>.
17. Marina I., Morato J., Rodriguez G., Mauri S., and Garcia S. ‘Nosocomial Weight Bias: Are There Differences between Medical Doctors and Surgeons’. *Obesity Reviews*, European and International Congress on Obesity, ECOICO 2020. Virtual., 21, no. SUPPL 1 (2020). <https://doi.org/10.1111/obr.13118>.
18. McLean, ME, LE McLean, AC McLean-Holden, LF Campbell, AM Horner, ML Kulkarni, LD Melville, and EA Fernandez. ‘Interphysician Weight Bias: A Cross-Sectional Observational Survey Study to Guide Implicit Bias Training in the Medical Workplace’. *ACADEMIC EMERGENCY MEDICINE* 28, no. 9 (September 2021): 1024–34. <https://doi.org/10.1111/acem.14269>.
19. Nickel, Felix, Christian Tapking, Laura Benner, Svenja Schüler, Gregor B Ottawa, Katja Krug, Beat P Müller-Stich, and Lars Fischer. ‘Video Teaching Leads to Improved Attitudes Towards Obesity-a Randomized Study with 949 Participants.’ *Obesity Surgery* 29, no. 7 (July 2019): 2078–86. <https://doi.org/10.1007/s11695-019-03804-9>.
20. Pearl, RL, D Argueso, and TA Wadden. ‘Effects of Medical Trainees’ Weight-Loss History on Perceptions of Patients with Obesity’. *MEDICAL EDUCATION* 51, no. 8 (August 2017): 802–11. <https://doi.org/10.1111/medu.13275>.
21. Poon M and Tarrant M. ‘Obesity: Attitudes of Undergraduate Student Nurses and Registered Nurses.’ *Journal of Clinical Nursing (Wiley-Blackwell)* 18, no. 16 (15 August 2009): 2355–65. <https://doi.org/10.1111/j.1365-2702.2008.02709.x>.
22. Popeski N., Kuzio C., Fowler M., Butalia S., and O’connell P. ‘Incorporating Obesity and Weight Bias Training Improved Attitudes of Hospital Staff Towards People With Obesity in Alberta’. *Canadian Journal of Diabetes*, 2021 Virtual Diabetes Canada/CSEM Professional Conference. Virtual, Online., 45, no. 7 Supplement (2021): S12. <https://doi.org/10.1016/j.jcjd.2021.09.039>.
23. Robinson B.B.E., Bacon J.G., & O’Reilly J. (1993). Fat phobia: Measuring, understanding, and changing anti-fat attitudes. *International Journal of Eating Disorders*, *14*(4), 467–480. Embase.
24. Robstad, N, F Siebler, U Soderhamn, T Westergren, and L Fegran. ‘Design and Psychometric Testing of Instruments to Measure Qualified Intensive Care Nurses’ Attitudes toward Obese Intensive Care Patients’. *RESEARCH IN NURSING & HEALTH* 41, no. 6 (December 2018): 525–34. <https://doi.org/10.1002/nur.21914>.
25. Robstad, N, T Westergren, F Siebler, U Soderhamn, and L Fegran. ‘Intensive Care Nurses’ Implicit and Explicit Attitudes and Their Behavioural Intentions towards Obese Intensive Care Patients’. *JOURNAL OF ADVANCED NURSING* 75, no. 12 (December 2019): 3631–42. <https://doi.org/10.1111/jan.14205>.
26. Sabin, Janice A, Maddalena Marini, and Brian A Nosek. ‘Implicit and Explicit Anti-Fat Bias among a Large Sample of Medical Doctors by BMI, Race/Ethnicity and Gender.’ *PloS One* 7, no. 11 (2012): e48448. <https://doi.org/10.1371/journal.pone.0048448>.
27. Schwartz, MB, HO Chambliss, KD Brownell, SN Blair, and C Billington. ‘Weight Bias among Health Professionals Specializing in Obesity’. *OBESITY RESEARCH* 11, no. 9 (September 2003): 1033–39. <https://doi.org/10.1038/oby.2003.142>.
28. Schwenke, M, M Luppa, A Pabst, FD Welzel, M Lobner, C Luck-Sikorski, A Kersting, M Bluher, and SG Riedel-Heller. ‘Attitudes and Treatment Practice of General Practitioners towards Patients with Obesity in Primary Care’. *BMC FAMILY PRACTICE* 21, no. 1 (17 August 2020). <https://doi.org/10.1186/s12875-020-01239-1>.
29. Setchell J., Watson B., Jones L., and Gard M. ‘Weight Stigma in Physiotherapy Practice: Patient Perceptions of Interactions with Physiotherapists’. *Manual Therapy* 20, no. 6 (2015): 835–41. <https://doi.org/10.1016/j.math.2015.04.001>.
30. Seymour, Justine, Jennifer L Barnes, Julie Schumacher, and Rachel L Vollmer. ‘A Qualitative Exploration of Weight Bias and Quality of Health Care Among Health Care Professionals Using Hypothetical Patient Scenarios.’ *Inquiry : A Journal of Medical Care Organization, Provision and Financing* 55 (January 2018): 46958018774171. <https://doi.org/10.1177/0046958018774171>.
31. Sherf-Dagan, Shiri, Lihi Schechter, Rita Lapidus, Nasser Sakran, David Goitein, and Asnat Raziel. ‘Perceptions of Success in Bariatric Surgery: A Nationwide Survey Among Medical Professionals.’ *Obesity Surgery* 28, no. 1 (January 2018): 135–41. <https://doi.org/10.1007/s11695-017-2800-9>.
32. Sikorski, C, M Luppa, H Glaesmer, E Braehler, HH Konig, and SG Riedel-Heller. ‘Attitudes of Health Care Professionals towards Female Obese Patients’. *OBESITY FACTS* 6, no. 6 (December 2013): 512–22. <https://doi.org/10.1159/000356692>.
33. Styk, W, E Wojtowicz, and S Zmorzynski. ‘Reliable Knowledge about Obesity Risk, Rather Than Personality, Is Associated with Positive Beliefs towards Obese People: Investigating Attitudes and Beliefs about Obesity, and Validating the Polish Versions of ATOP, BAOP and ORK-10 Scales’. *INTERNATIONAL JOURNAL OF ENVIRONMENTAL RESEARCH AND PUBLIC HEALTH* 19, no. 22 (November 2022). <https://doi.org/10.3390/ijerph192214977>.
34. Swift, J., Glazebrook, C., & Macdonald, I. (2006). Validation of a brief, reliable scale to measure knowledge about the health risks associated with obesity. *INTERNATIONAL JOURNAL OF OBESITY*, *30*(4), 661–668. <https://doi.org/10.1038/sj.ijo.0803165>
35. Teachman, B A, and K D Brownell. ‘Implicit Anti-Fat Bias among Health Professionals: Is Anyone Immune?’ *International Journal of Obesity and Related Metabolic Disorders : Journal of the International Association for the Study of Obesity* 25, no. 10 (October 2001): 1525–31. <https://doi.org/10.1038/sj.ijo.0801745>.
36. Tomiyama, A Janet, Laura E Finch, Angela C Incollingo Belsky, Julia Buss, Carrie Finley, Marlene B Schwartz, and Jennifer Daubenmier. ‘Weight Bias in 2001 versus 2013: Contradictory Attitudes among Obesity Researchers and Health Professionals.’ *Obesity (19307381)* 23, no. 1 (January 2015): 46–53. <https://doi.org/10.1002/oby.20910>.
37. Tsai A.G., Histon T., Kyle T.K., Rubenstein N., and Donahoo W.T. ‘Evidence of a Gap in Understanding Obesity among Physicians’. *Obesity Science and Practice* 4, no. 1 (2018): 46–51. <https://doi.org/10.1002/osp4.146>.
38. Vallis TM, Currie B, Lawlor D, and Ransom T. ‘Healthcare Professional Bias against the Obese: How Do We Know If We Have a Problem?’ *Canadian Journal of Diabetes* 31, no. 4 (December 2007): 365–70.
39. Wang, Y, Y Ding, DP Song, DQ Zhu, and JR Wang. ‘Attitudes Toward Obese Persons and Weight Locus of Control in Chinese Nurses A Cross-Sectional Survey’. *NURSING RESEARCH* 65, no. 2 (March 2016): 126–31. <https://doi.org/10.1097/NNR.0000000000000145>.
40. Wise, Frances M., Darren W. Harris, and John H. Olver. ‘Attitudes to Obesity Among Rehabilitation Health Professionals in Australia.’ *Journal of Allied Health* 43, no. 3 (2014): 162–68.
41. Wolf, Corri. ‘Physician Assistant Students’ Attitudes About Obesity and Obese Individuals.’ *Journal of Physician Assistant Education (Physician Assistant Education Association)* 21, no. 4 (December 2010): 37–40. <https://doi.org/10.1097/01367895-201021040-00005>.
42. Wötzel, Hendrik, Claudia Sikorski, Georg Schomerus, Melanie Luppa, and Steffi G Riedel-Heller. ‘[Weight Related Stigmatisation of Obese Individuals in Somatic and Mental Health Care - Are There Differences?].’ *Psychiatrische Praxis* 42, no. 6 (September 2015): 328–32. <https://doi.org/10.1055/s-0034-1370097>.
43. Wynn T., Islam N., Thompson C., and Myint K.S. ‘The Effect of Knowledge on Healthcare Professionals’ Perceptions of Obesity’. *Obesity Medicine* 11, no. (Wynn, Islam, Thompson, Myint) Norwich Medical School, University of East Anglia, Norwich NR4 7TJ, United Kingdom (2018): 20–24. <https://doi.org/10.1016/j.obmed.2018.06.006>.
44. Yılmaz, Hacı Ömer, and Nurcan Yabancı Ayhan. ‘Is There Prejudice against Obese Persons among Health Professionals? A Sample of Student Nurses and Registered Nurses.’ *Perspectives in Psychiatric Care* 55, no. 2 (April 2019): 262–68. <https://doi.org/10.1111/ppc.12359>.
45. Zagaria, A, E Mocini, S Cerolini, LM Donini, G Castelnuovo, GM Manzoni, G Pietrabissa, and C Lombardo. ‘A Validation Study of the Italian Version of the Attitudes Toward Obese Persons (I-ATOP) Questionnaire’. *OBESITY RESEARCH & CLINICAL PRACTICE* 16, no. 3 (May 2022): 262–68. <https://doi.org/10.1016/j.orcp.2022.05.004>.

**Articles removed for full article review: 12**

- **No full article available (Reached out to authors) = 5**
- **No use of OM = 3**
- **Not HCP Population = 2**
- **Validation of Italian version – non-English OM 2**

**Total Full Articles Reviewed: 33**

**Full Articles for Review: = 33 – See attached summary table**

**1. Instrument Development Literature**

1. Akman M., Kivrakoglu E., Cifcili S., and Unalan P.C. ‘Weight Bias among Primary Care Health Professionals: Personal Attitude Matters’. *Obesity and Metabolism* 6, no. 2–3 (2010): 63–68.
2. ALLISON, D., BASILE, V., & YUKER, H. (1991). THE MEASUREMENT OF ATTITUDES TOWARD AND BELIEFS ABOUT OBESE PERSONS. *INTERNATIONAL JOURNAL OF EATING DISORDERS*, *10*(5), 599–607.
3. Bacon, J., Scheltema, K., & Robinson, B. (2001). Fat phobia scale revisited: The short form. *INTERNATIONAL JOURNAL OF OBESITY*, *25*(2), 252–257. <https://doi.org/10.1038/sj.ijo.0801537>
4. Cain, P., Donaghue, N., & Ditchburn, G. (2022). Development and validation of the Fat Attitudes Assessment Toolkit (FAAT): A multidimensional nonstigmatizing measure of contemporary attitudes toward fatness and fat people. *JOURNAL OF APPLIED SOCIAL PSYCHOLOGY*, *52*(12), 1121–1145. <https://doi.org/10.1111/jasp.12882>
5. CRANDALL, C. (1994). PREJUDICE AGAINST FAT PEOPLE - IDEOLOGY AND SELF-INTEREST. *JOURNAL OF PERSONALITY AND SOCIAL PSYCHOLOGY*, *66*(5), 882–894. <https://doi.org/10.1037/0022-3514.66.5.882>
6. Lewis, R., Cash, T., Jacobi, L., & BubbLewis, C. (1997). Prejudice toward fat people: The development and validation of the antifat attitudes test. *OBESITY RESEARCH*, *5*(4), 297–307. <https://doi.org/10.1002/j.1550-8528.1997.tb00555.x>
7. Robinson B.B.E., Bacon J.G., & O’Reilly J. (1993). Fat phobia: Measuring, understanding, and changing anti-fat attitudes. *International Journal of Eating Disorders*, *14*(4), 467–480. Embase.
8. Swift, J., Glazebrook, C., & Macdonald, I. (2006). Validation of a brief, reliable scale to measure knowledge about the health risks associated with obesity. *INTERNATIONAL JOURNAL OF OBESITY*, *30*(4), 661–668. <https://doi.org/10.1038/sj.ijo.0803165>
9. Teachman, B A, and K D Brownell. ‘Implicit Anti-Fat Bias among Health Professionals: Is Anyone Immune?’ *International Journal of Obesity and Related Metabolic Disorders : Journal of the International Association for the Study of Obesity* 25, no. 10 (October 2001): 1525–31. <https://doi.org/10.1038/sj.ijo.0801745>.

**2. Literature utilising Instrument**

1. Bøker Lund, Thomas, John Brodersen, and Peter Sandøe. ‘A Study of Anti-Fat Bias among Danish General Practitioners and Whether This Bias and General Practitioners’ Lifestyle Can Affect Treatment of Tension Headache in Patients with Obesity.’ *Obesity Facts* 11, no. 6 (2018): 501–13. <https://doi.org/10.1159/000493373>.
2. Da Q Zhu, Ian J. Norman, and Alison E. While. ‘Nurses’ Self-Efficacy and Practices Relating to Weight Management of Adult Patients: A Path Analysis.’ *International Journal of Behavioral Nutrition & Physical Activity* 10 (January 2013): 131–53. <https://doi.org/10.1186/1479-5868-10-131>.
3. Darling, Rosa, and A. Serdar Atav. ‘Attitudes toward Obese People: A Comparative Study of Nursing, Education, and Social Work Students’. *Journal of Professional Nursing* 35, no. 2 (March 2019): 138–46. <https://doi.org/10.1016/j.profnurs.2018.07.009>.
4. Diversi, T M, R Hughes, and K J Burke. ‘The Prevalence and Practice Impact of Weight Bias amongst Australian Dietitians.’ *Obesity Science & Practice* 2, no. 4 (December 2016): 456–65. <https://doi.org/10.1002/osp4.83>.
5. Elboim-Gabyzon, M, K Attar, and S Peleg. ‘Weight Stigmatization among Physical Therapy Students and Registered Physical Therapists’. *OBESITY FACTS* 13, no. 2 (May 2020): 104–16. <https://doi.org/10.1159/000504809>.
6. Gujral, Harpreet, Christine Tea, and Michael Sheridan. ‘Evaluation of Nurse’s Attitudes toward Adult Patients of Size.’ *Surgery for Obesity and Related Diseases : Official Journal of the American Society for Bariatric Surgery* 7, no. 4 (July 2011): 536–40. <https://doi.org/10.1016/j.soard.2011.03.008>.
7. Jones C.A. and Forhan M. ‘Addressing Weight Bias and Stigma of Obesity amongst Physiotherapists’. *Physiotherapy Theory and Practice*, no. (Jones, Forhan) Faculty of Rehabilitation Medicine, University of Alberta, 2-50 Corbett Hall, Edmonton, AB, Canada (2019): 1–9. <https://doi.org/10.1080/09593985.2019.1648623>.
8. Khandalavala B.N., Rojanala A., Geske J.A., Koran-Scholl J.B., and Guck T.P. ‘Obesity Bias in Primary Care Providers’. *Family Medicine* 46, no. 7 (2014): 532–35.
9. McLean, ME, LE McLean, AC McLean-Holden, LF Campbell, AM Horner, ML Kulkarni, LD Melville, and EA Fernandez. ‘Interphysician Weight Bias: A Cross-Sectional Observational Survey Study to Guide Implicit Bias Training in the Medical Workplace’. *ACADEMIC EMERGENCY MEDICINE* 28, no. 9 (September 2021): 1024–34. <https://doi.org/10.1111/acem.14269>.
10. Nickel, Felix, Christian Tapking, Laura Benner, Svenja Schüler, Gregor B Ottawa, Katja Krug, Beat P Müller-Stich, and Lars Fischer. ‘Video Teaching Leads to Improved Attitudes Towards Obesity-a Randomized Study with 949 Participants.’ *Obesity Surgery* 29, no. 7 (July 2019): 2078–86. <https://doi.org/10.1007/s11695-019-03804-9>.
11. Pearl, RL, D Argueso, and TA Wadden. ‘Effects of Medical Trainees’ Weight-Loss History on Perceptions of Patients with Obesity’. *MEDICAL EDUCATION* 51, no. 8 (August 2017): 802–11. <https://doi.org/10.1111/medu.13275>.
12. Poon M and Tarrant M. ‘Obesity: Attitudes of Undergraduate Student Nurses and Registered Nurses.’ *Journal of Clinical Nursing (Wiley-Blackwell)* 18, no. 16 (15 August 2009): 2355–65. <https://doi.org/10.1111/j.1365-2702.2008.02709.x>.
13. Robstad, N, F Siebler, U Soderhamn, T Westergren, and L Fegran. ‘Design and Psychometric Testing of Instruments to Measure Qualified Intensive Care Nurses’ Attitudes toward Obese Intensive Care Patients’. *RESEARCH IN NURSING & HEALTH* 41, no. 6 (December 2018): 525–34. <https://doi.org/10.1002/nur.21914>.
14. Robstad, N, T Westergren, F Siebler, U Soderhamn, and L Fegran. ‘Intensive Care Nurses’ Implicit and Explicit Attitudes and Their Behavioural Intentions towards Obese Intensive Care Patients’. *JOURNAL OF ADVANCED NURSING* 75, no. 12 (December 2019): 3631–42. <https://doi.org/10.1111/jan.14205>.
15. Sabin, Janice A, Maddalena Marini, and Brian A Nosek. ‘Implicit and Explicit Anti-Fat Bias among a Large Sample of Medical Doctors by BMI, Race/Ethnicity and Gender.’ *PloS One* 7, no. 11 (2012): e48448. <https://doi.org/10.1371/journal.pone.0048448>.
16. Schwartz, MB, HO Chambliss, KD Brownell, SN Blair, and C Billington. ‘Weight Bias among Health Professionals Specializing in Obesity’. *OBESITY RESEARCH* 11, no. 9 (September 2003): 1033–39. <https://doi.org/10.1038/oby.2003.142>.
17. Schwenke, M, M Luppa, A Pabst, FD Welzel, M Lobner, C Luck-Sikorski, A Kersting, M Bluher, and SG Riedel-Heller. ‘Attitudes and Treatment Practice of General Practitioners towards Patients with Obesity in Primary Care’. *BMC FAMILY PRACTICE* 21, no. 1 (17 August 2020). <https://doi.org/10.1186/s12875-020-01239-1>.
18. Seymour, Justine, Jennifer L Barnes, Julie Schumacher, and Rachel L Vollmer. ‘A Qualitative Exploration of Weight Bias and Quality of Health Care Among Health Care Professionals Using Hypothetical Patient Scenarios.’ *Inquiry : A Journal of Medical Care Organization, Provision and Financing* 55 (January 2018): 46958018774171. <https://doi.org/10.1177/0046958018774171>.
19. Sikorski, C, M Luppa, H Glaesmer, E Braehler, HH Konig, and SG Riedel-Heller. ‘Attitudes of Health Care Professionals towards Female Obese Patients’. *OBESITY FACTS* 6, no. 6 (December 2013): 512–22. <https://doi.org/10.1159/000356692>.
20. Vallis TM, Currie B, Lawlor D, and Ransom T. ‘Healthcare Professional Bias against the Obese: How Do We Know If We Have a Problem?’ *Canadian Journal of Diabetes* 31, no. 4 (December 2007): 365–70.
21. Wang, Y, Y Ding, DP Song, DQ Zhu, and JR Wang. ‘Attitudes Toward Obese Persons and Weight Locus of Control in Chinese Nurses A Cross-Sectional Survey’. *NURSING RESEARCH* 65, no. 2 (March 2016): 126–31. <https://doi.org/10.1097/NNR.0000000000000145>.
22. Wise, Frances M., Darren W. Harris, and John H. Olver. ‘Attitudes to Obesity Among Rehabilitation Health Professionals in Australia.’ *Journal of Allied Health* 43, no. 3 (2014): 162–68.
23. Wynn T., Islam N., Thompson C., and Myint K.S. ‘The Effect of Knowledge on Healthcare Professionals’ Perceptions of Obesity’. *Obesity Medicine* 11, no. (Wynn, Islam, Thompson, Myint) Norwich Medical School, University of East Anglia, Norwich NR4 7TJ, United Kingdom (2018): 20–24. <https://doi.org/10.1016/j.obmed.2018.06.006>.
24. Yılmaz, Hacı Ömer, and Nurcan Yabancı Ayhan. ‘Is There Prejudice against Obese Persons among Health Professionals? A Sample of Student Nurses and Registered Nurses.’ *Perspectives in Psychiatric Care* 55, no. 2 (April 2019): 262–68. <https://doi.org/10.1111/ppc.12359>.

**Articles that fulfil the inclusion criteria for COSMIN Review = 4**

1. Akman M., Kivrakoglu E., Cifcili S., and Unalan P.C. ‘Weight Bias among Primary Care Health Professionals: Personal Attitude Matters’. *Obesity and Metabolism* 6, no. 2–3 (2010): 63–68.
2. Robinson B.B.E., Bacon J.G., & O’Reilly J. (1993). Fat phobia: Measuring, understanding, and changing anti-fat attitudes. *International Journal of Eating Disorders*, *14*(4), 467–480. Embase.
3. Swift, J., Glazebrook, C., & Macdonald, I. (2006). Validation of a brief, reliable scale to measure knowledge about the health risks associated with obesity. *INTERNATIONAL JOURNAL OF OBESITY*, *30*(4), 661–668. <https://doi.org/10.1038/sj.ijo.0803165>
4. Teachman, B A, and K D Brownell. ‘Implicit Anti-Fat Bias among Health Professionals: Is Anyone Immune?’ *International Journal of Obesity and Related Metabolic Disorders : Journal of the International Association for the Study of Obesity* 25, no. 10 (October 2001): 1525–31. <https://doi.org/10.1038/sj.ijo.0801745>.

**Updated Search to align with the previous review 06/02/2023 to 01/05/2025**

**Health Professional-related Databases utilised :**

- Embase
- Medline
- Web of Science (core collection)
- Psychc NFO
- CINAHL (complete)
- Google Scholar

| **CINAHL via EBSCO**  **Attitudes and Beliefs**  **Search 4** | obesity or overweight or fat or obese or unhealthy weight or high bmi  AND attitudes or perceptions or opinions or thoughts or feelings or beliefs or views or attitudes towards  AND adults or adult or aged or elderly or middle aged or older person or young adult  AND outcome measure or measure or instrument or questionnaire or assessment or evaluation or scale or test or tool  AND clinicians or health professionals or nurses or physicians or doctors or physiotherapists or physical therapists or allied health or therapist  AND psychometrics or validity or reliability or psychometric properties | 4 | Import to Zotero. | 06/02/2023 |
| --- | --- | --- | --- | --- |
| **MEDLINE via EBSCO**  **Attitudes and Beliefs**  **Search 4** | obesity or overweight or fat or obese or unhealthy weight or high bmi  AND attitudes or perceptions or opinions or thoughts or feelings or beliefs or views or attitudes towards  AND adults or adult or aged or elderly or middle aged or older person or young adult  AND outcome measure or measure or instrument or questionnaire or assessment or evaluation or scale or test or tool  AND clinicians or health professionals or nurses or physicians or doctors or physiotherapists or physical therapists or allied health or therapist  AND psychometrics or validity or reliability or psychometric properties | 4 | Import to Zotero. | 06/02/2023 |
| **APA PsycINFO via EBSCO**  **Attitudes and Beliefs**  **Search 4** | obesity or overweight or fat or obese or unhealthy weight or high bmi  AND attitudes or perceptions or opinions or thoughts or feelings or beliefs or views or attitudes towards  AND adults or adult or aged or elderly or middle aged or older person or young adult  AND outcome measure or measure or instrument or questionnaire or assessment or evaluation or scale or test or tool  AND clinicians or health professionals or nurses or physicians or doctors or physiotherapists or physical therapists or allied health or therapist  AND psychometrics or validity or reliability or psychometric properties | 0 |  | 06/02/2023 |
| **EMBASE**  **Attitudes and Beliefs**  **Search 4** | obesity or overweight or fat or obese or unhealthy weight or high bmi  AND attitudes or perceptions or opinions or thoughts or feelings or beliefs or views or attitudes towards  AND adults or adult or aged or elderly or middle aged or older person or young adult  AND outcome measure or measure or instrument or questionnaire or assessment or evaluation or scale or test or tool  AND clinicians or health professionals or nurses or physicians or doctors or physiotherapists or physical therapists or allied health or therapist  AND psychometrics or validity or reliability or psychometric properties | 11 | Import to Zotero. | 06/02/2023 |
| **WEB OF SCIENCE**  **Attitudes and Beliefs**  **Search 4** | obesity or overweight or fat or obese or unhealthy weight or high bmi  AND attitudes or perceptions or opinions or thoughts or feelings or beliefs or views or attitudes towards  AND adults or adult or aged or elderly or middle aged or older person or young adult  AND outcome measure or measure or instrument or questionnaire or assessment or evaluation or scale or test or tool  AND clinicians or health professionals or nurses or physicians or doctors or physiotherapists or physical therapists or allied health or therapist  AND psychometrics or validity or reliability or psychometric properties | 50 | Import to Zotero. | 06/02/2023 |

| **CINAHL via EBSCO**  **Knowledge**  **Search 4** | obesity or overweight or fat or obese or unhealthy weight or high bmi  AND knowledge or education or understanding or awareness or training or skill AND adults or adult or aged or elderly or middle aged or older person or young adult  AND outcome measure or measure or instrument or questionnaire or assessment or evaluation or scale or test or tool  AND clinicians or health professionals or nurses or physicians or doctors or physiotherapists or physical therapists or allied health or therapist  AND psychometrics or validity or reliability or psychometric properties | 4 | Import to Zotero. | 06/02/2023 |
| --- | --- | --- | --- | --- |
| **MEDLINE via EBSCO**  **Knowledge**  **Search 4** | obesity or overweight or fat or obese or unhealthy weight or high bmi  AND knowledge or education or understanding or awareness or training or skill AND adults or adult or aged or elderly or middle aged or older person or young adult  AND outcome measure or measure or instrument or questionnaire or assessment or evaluation or scale or test or tool  AND clinicians or health professionals or nurses or physicians or doctors or physiotherapists or physical therapists or allied health or therapist  AND psychometrics or validity or reliability or psychometric properties | 15 | Import to Zotero. | 06/02/2023 |
| **APA PsycINFO via EBSCO**  **Knowledge**  **Search 4** | obesity or overweight or fat or obese or unhealthy weight or high bmi  AND knowledge or education or understanding or awareness or training or skill AND adults or adult or aged or elderly or middle aged or older person or young adult  AND outcome measure or measure or instrument or questionnaire or assessment or evaluation or scale or test or tool  AND clinicians or health professionals or nurses or physicians or doctors or physiotherapists or physical therapists or allied health or therapist  AND psychometrics or validity or reliability or psychometric properties | 3 | Import to Zotero. | 06/02/2023 |
| **EMBASE**  **Knowledge**  **Search 4** | obesity or overweight or fat or obese or unhealthy weight or high bmi  AND knowledge or education or understanding or awareness or training or skill AND adults or adult or aged or elderly or middle aged or older person or young adult  AND outcome measure or measure or instrument or questionnaire or assessment or evaluation or scale or test or tool  AND clinicians or health professionals or nurses or physicians or doctors or physiotherapists or physical therapists or allied health or therapist  AND psychometrics or validity or reliability or psychometric properties | 20 | Import to Zotero. | 06/02/2023 |
| **WEB OF SCIENCE**  **Knowledge**  **Search 4** | obesity or overweight or fat or obese or unhealthy weight or high bmi  AND knowledge or education or understanding or awareness or training or skill AND adults or adult or aged or elderly or middle aged or older person or young adult  AND outcome measure or measure or instrument or questionnaire or assessment or evaluation or scale or test or tool  AND clinicians or health professionals or nurses or physicians or doctors or physiotherapists or physical therapists or allied health or therapist  AND psychometrics or validity or reliability or psychometric properties | 268 | Import to Zotero. | 06/02/2023 |

**01/05/2025**

- Total Number of Search items imported into Zotero = 379
- Total Number of Duplicates and non-journal articles removed = 295
- Total Abstracts for review = 84

Total papers identified in the Search for full review that weren’t identified in the previous searches = 0

**WENT BACK THROUGH REJECTIONS TO DOUBLE CHECK – NO FURTHER ABSTRACTS IDENTIFIED.** – 01/05/2025

**Common Themes for exclusion –**

- no clear/ specific outcome measurement used/ developed to assess attitudes, beliefs, or knowledge of obesity.
- non-HPC populations – general population or university students
