# Supplementary material for: A Scoping Review of Outcome Measurement Instruments Assessing Healthcare Professionals’ Knowledge, Attitudes and Beliefs About Adult Obesity
Source: J Obes. 2026 Aug 13;2026:1363752. doi: 10.1155/jobe/1363752 (PMC13470226; doi:10.1155/jobe/1363752)
Supplement: Supplementary file 2 [file JOBE-2026-1363752-s001.zip › Supplementary Table 2.docx]

| **Name of instrument** | **Developer** | **Year of Development / Publication** | **Construct (s)** | **Country of development (Language)** | **Targeted population** | **Healthcare Professional Type (Actual Sample)** | **Healthcare Professional Involvement in Instrument Development (Yes/ No)** | **Mode of administration (e.g., self-report)** | **(Sub)scale(s)/Content(s) (number of items)** | **Response options** | **Range of scores/scoring** |
| --- | --- | --- | --- | --- | --- | --- | --- | --- | --- | --- | --- |
| **Implicit Anti-Fat Bias Instrument** | Greenwald et al., (1998) developed the original Implicit Association Test (IAT), which was later adapted for obesity by Teachman and Brownell (2001). | 2001  *Development year not explicitly stated; publication year used as a proxy* | Weight Attitudes | USA | Healthcare Professionals involved in obesity care and treatment | Physicians (72%), nutritionists, pharmacists, and “other” obesity focused Healthcare Professionals' | No – Healthcare Professional participants only, no development input noted | Timed computer-based reaction-time task (Implicit Association Test)  *(Not self-reported)* | 2 distinct tasks:  1. Measure attitudes  2. Measure stereotype.  Each IAT task consisted of two pages:  Page One: The target and attribute categories were paired to match negative automatic associations with overweight.  Page Two: The target and attribute categories were paired to contradict expected associations. | Classify as many words as possible on each page | Composite Scoring:  Positive effects indicate anti-fat or pro-thin bias.  Negative effects indicate anti-thin or pro-fat bias |
| **Explicit fat-thin bias** | Teachman *and Brownell (2001) - used adapted self-reported scales.* | 2001  *Development year not explicitly stated; publication year used as a proxy* | Weight Attitudes | USA | Healthcare Professionals involved in obesity care and treatment | Physicians (72%), nutritionists, pharmacists, and “other” obesity focused Healthcare Professionals' | No – Healthcare Professional participants only, no development input noted | Self-Reported | 1. To assess explicit attitudes, participants were asked to rate their feelings about ‘fat people’ and their feelings about ‘thin people’ as bad vs good  2. To assess beliefs about fat and thin people as lazy vs motivated. | Seven-point semantic differential scales with 1 = bad/negative rating and 7 = good/positive rating. | A difference score between the two items was calculated to evaluate attitudes toward fat people relative to thin people  A score above the neutral point on the scale (ie greater than 4) indicates anti-fat bias, while a score <4. indicates pro-thin bias |
| **Fat Phobia Scale (FPS)** | *Robinson (1984); published by Robinson B.B.E., Bacon J.G., & O’Reilly J. (1993).* | 1984(development); 1993 (publication)  *Development year reported by authors; publication year shown for transparency* | Fat Phobia/ negative attitudes toward fat people | USA | General adult population; *not designed specifically for healthcare professionals.* | HCP subgroup from study 1; mental health and medical professionals (31%), and other professionals, students and general public participants | No reported involvement from healthcare professionals  (Originally designed for the general population) | Self-reported | 50-items | 5-point semantic differential scale | The total of the scores on the scale is added up and divided by the number of items (50)  The score is converted to the original 5-point scale (1-5).  Low Score = less fat phobia  High Score = more fat phobia. |
| **Obesity Risk Knowledge Scale (ORK-10)** | Swift, Glazebrook & MacDonald (2006) | 2006  *Development year not explicitly stated; publication year used as a proxy* | Explicit knowledge regarding the health risks associated with obesity | UK | General public and selected at-risk groups; *not specifically developed for healthcare professionals* | Obesity-related experts (Association for the Study of Obesity members) | No reported healthcare professional involvement in instrument development. | Self-reported | 10-item instrument | ‘True’ = 1 point  ‘False’ = zero points  ‘Don’t know’ = zero points | Scores on the ORK-10 scale range between zero and 10.  Higher scores indicate higher levels of knowledge |
| **Obesity Perception Survey** | *Akman et al. 2010* | 2010  *Development year not explicitly stated; publication year used as a proxy* | Weight Bias | Turkey | Primary care Healthcare Professionals (Physicians and nurses) | Primary care physicians (50.9%) and nurses (49.1%) in Istanbul primary care setting | Pilot tested with 10 healthcare professionals but no reported involvement in instrument development. | Self-reported | 24-item instrument  2 x subgroups: personal attitudes & professional practice patterns | 5-choice Likert type scale  (0: totally disagree, to 5: totally agree) | Those that rated 4 and 5 in scale were grouped as “those who agreed with the statement”  The other ratings as “those who disagreed with the statement”  Higher scores showed more weight bias |
